# Optimization of the Appearance Quality in CO_2_ Processed Ready-to-Eat Carrots through Image Analysis

**DOI:** 10.3390/foods10122999

**Published:** 2021-12-04

**Authors:** Gianmarco Barberi, Víctor González-Alonso, Sara Spilimbergo, Massimiliano Barolo, Alessandro Zambon, Pierantonio Facco

**Affiliations:** 1 CAPE-Lab–Computer Aided Process Engineering Laboratory, Department of Industrial Engineering, University of Padova, Via Marzolo, 9-35131 Padova, Italy; gianmarco.barberi@phd.unipd.it (G.B.); max.barolo@unipd.it (M.B.); 2 Superunit–CO2 Innovation Lab, Department of Industrial Engineering, University of Padova, Via Marzolo, 9-35131 Padova, Italy; victor.glez1994@gmail.com (V.G.-A.); sara.spilimbergo@unipd.it (S.S.); alessandro.zambon@unipd.it (A.Z.)

**Keywords:** high-pressure CO_2_, food appearance, ready-to-eat carrots, food preservation, modified atmosphere packaging, image analysis, multivariate hypothesis testing

## Abstract

A high-pressure CO_2_ process applied to ready-to-eat food products guarantees an increase of both their microbial safety and shelf-life. However, the treatment often produces unwanted changes in the visual appearance of products depending on the adopted process conditions. Accordingly, the alteration of the visual appearance influences consumers’ perception and acceptability. This study aims at identifying the optimal treatment conditions in terms of visual appearance by using an artificial vision system. The developed methodology was applied to fresh-cut carrots (*Daucus carota*) as the test product. The results showed that carrots packaged in 100% CO_2_ and subsequently treated at 6 MPa and 40 °C for 15 min maintained an appearance similar to the fresh product for up to 7 days of storage at 4 °C. Mild appearance changes were identified at 7 and 14 days of storage in the processed products. Microbiological analysis performed on the optimal treatment condition showed the microbiological stability of the samples up to 14 days of storage at 4 °C. The artificial vision system, successfully applied to the CO_2_ pasteurization process, can easily be applied to any food process involving changes in the appearance of any food product.

## 1. Introduction

In the last decade, the consumption of ready-to-eat (RTE) products has widely increased. Among the RTE products, such as fresh-and-cut fruits and vegetables, carrots’ market increased by 5.5% between 2020 and 2021, being the 5th most produced vegetable in the US in 2020 [1]. Raw RTE carrots are usually packaged in a modified atmosphere packaging (MAP) which extends the storage time and increases the product shelf life. Due to the recent outbreaks in RTE foods [2,3], microbial safety is of primary importance and new strategies should be implemented to reduce the risk of food contamination. Recently, MAP has been coupled with innovative low temperature treatments to increase the microbiological safety, such as gamma radiation [4], ultraviolet light [5], ozone [6], high-voltage electrostatic fields [7], and high pressure [8]. Spilimbergo et al. [9] proposed a new method to inactivate the microbial population in food at low temperature which combines the advantages of supercritical CO_2_ inactivation within a MAP (ScCO_2_-MAP). Similar to traditional supercritical CO_2_ (ScCO_2_) treatment [10,11], ScCO_2_-MAP uses ScCO_2_ to inactivate microorganisms, but acts directly inside the packaging, thus reducing the risk of cross-contamination after processing. 

The adoption of novel preservation technologies by industry has to comply not only with the microbial safety of the food, but also with the maintenance of fresh food characteristics. Indeed, the sensorial characteristics of a RTE product, such as visual appearance, texture, moisture content, and flavor, are the major driving factors affecting sensory perception and consumer preferences [12,13]. In this context, high-pressure technologies affect the texture and color of the products and these effects depend on the adopted processing conditions and the specific product [14]. Fast, cheap, and straightforward methods to identify the optimal processing conditions which preserve and/or minimize the visual changes in the food are of paramount importance.

The visual appearance of food products is one of the most difficult parameters to assess in an objective and repeatable manner. The preservation of a fresh-like appearance is usually evaluated through color measurement [15], because color changes have a primary role in food acceptability and preference [16]. However, the analytical methodology for color measurement based on a colorimeter is time-consuming and suffers from several drawbacks, mostly related to the high sensitivity to color inhomogeneities in the surface of the inspected product [17]. Since the color measurements are performed in specific spots, they capture only local color information, completely disregarding information on the superficial structure of the food matrix. This structural information, captured by the spatial distribution of color over the food surface, is another important factor affecting consumers’ perception [18]. 

Artificial vision systems are effective tools for the evaluation of color changes in fruits, vegetables, and meat. In fact, image analysis is an accurate, repeatable, and inexpensive way to detect even minor differences in product appearance. Artificial vision systems have been used for color measurements [19,20], for the estimation of chemical parameters [21], for food authentication [22], and for defects identification [23]. For example, in recent years, artificial vision systems have been used to evaluate the quality and to detect contaminants in the soybean industry [24], to grade the maturity level of mangoes [25], and to evaluate the quality and freshness of saffron [26], while systems based on hyperspectral images have been used to detect defects and diseases of orange skin [27]. Artificial vision systems exploit the information on the color distribution over the product surface to significantly improve their recognition and detection capabilities [22,28]. However, exploiting the information on the changes in the color distribution over the food surface is not straightforward, and appropriate multivariate techniques [29] are required to deal with the large variability in food appearance. Despite the use of artificial vision systems being widespread in the food industry, to our knowledge, they have not been previously applied to food undergoing ScCO_2_ treatment in any form. The use of artificial vision systems in ScCO_2_ food treatments could provide a reliable methodology to characterize the effect of these innovative preservation technologies on the visual appearance quality of food products. Furthermore, artificial vision systems could be exploited to reduce the experimental burden of microbiological tests, allowing the microbiological inactivation to be verified only in the samples which meet the consumers’ acceptability criteria in terms of visual appearance. 

In this study we developed an artificial vision system to evaluate, through multivariate hypothesis testing, the appearance of the food after the ScCO_2_-MAP food preservation process is carried out. The methodology is applied on fresh-cut carrots (*Daucus carota*) and is aimed at identifying the optimal treatment conditions in terms of pressure, temperature, time, and %CO_2_ in the MAP to preserve a fresh-like appearance after processing and during storage (up to 14 days). In this way, the experimental campaign to verify the microbiological inactivation is considerably reduced and carried out in the restricted domain of the optimal process conditions identified by the artificial vision system as the ones that ensure a fresh-like product. Microbiological analysis is performed for the naturally present microorganisms (total mesophilic bacteria, yeasts, and molds) and inoculated *E. coli*.

## 2. Materials and Methods

In this study, the analyses are carried out in five steps: (i) preparation of the samples; (ii) definition of the experimental design; (iii) ScCO_2_-MAP treatment and storage; (iv) evaluation of the food appearance through the artificial vision system and identification of the optimal operating conditions to reduce the experimental domain for microbiological testing; and (v) execution of the microbiological testing on the optimal conditions identified by the artificial vision system and statistical analysis.

### 2.1. Sample Preparation and Storage

Fresh orange carrots (*Daucus carota*) harvested in mid-April, with approximatively the same dimensions and mature level, were purchased from a local farm market (Padova, Italy). Carrots were preliminary packaged by the vendor in plastic packages with no modified atmosphere and stored at 4 °C. After purchasing, carrots were uniformly stored at 4 °C and processed within 2 h. 

Carrots were cut into round slices of 3.25 ± 0.25 g weight, 3.00 ± 0.25 cm diameter, and 3 ± 1 mm thickness just before processing. After processing, carrots were stored at 4 °C up to 14 days to perform a storage test.

### 2.2. Experimental Desing

Carrots were packaged with three different MAP compositions: (i) 100% CO_2_ (carbon dioxide 4.0, purity > 99.8%, Rivoira, Milan, Italy) referred to as MAP1; (ii) 90% CO_2_ with 10% O_2_ (oxygen 2.0, purity >99.5%, Rivoira, Milan, Italy) referred to as MAP2; and (iii) air referred to as MAP3. MAP1 was selected to resemble the traditional ScCO_2_, which uses pure CO_2_; MAP2 was selected to study the effect of small O_2_ concentrations; and MAP3 was selected as the control.

The ScCO_2_-MAP treatment conditions were selected according to a 2^3−1^ fractional factorial design of experiments (DoE) [30] with three replicates of the central point. The entire set of treatment conditions defined by the DoE was carried out for each MAP composition in two replicates. Three factors affecting process performance were considered: process temperature, process pressure, and treatment time. Two factor levels were considered: 25 °C and 40 °C for temperature; 6 and 12 MPa for pressure; and 15 and 45 min for treatment time. Based on the preliminary tests, the ranges of the process operating conditions were chosen (i) to match typical treatment conditions that are effective for the preservation of foods using the traditional ScCO_2_ treatment [10,11,31,32] and do not significantly affect the food quality [14,33,34], and (ii) to test the effect on product appearance of both subcritical and supercritical CO_2_. The ScCO_2_-MAP treatment conditions tested in this study are reported in Table 1. 

At each treatment condition, four packages were treated simultaneously. Each package was analyzed at a different control time along the storage test performed up to 14 days at 4 °C and subsequently discarded. The control times were set to 0, 3, 7, and 14 days of storage. Control times were selected to analyze the appearance changes progressively along an average shelf life for carrots with more emphasis on the first week of storage. Consequently, in this study 168 carrot packages were prepared, treated, and analyzed (3 MAP compositions × (4 fractional factorial + 3 central) treatment conditions × 4 time controls × 2 replicates).

Untreated control samples were prepared for each MAP composition. The control samples’ preparation and storage were identical to that of all other samples, but they were packaged and stored without any treatment; they were analyzed at the same control times as treated samples. In this study, 9 control packages were prepared (3 MAP compositions × 3 time controls (3, 7, and 14 days)).

### 2.3. High-Pressure CO_2_ Treatment

**Figure 1:**
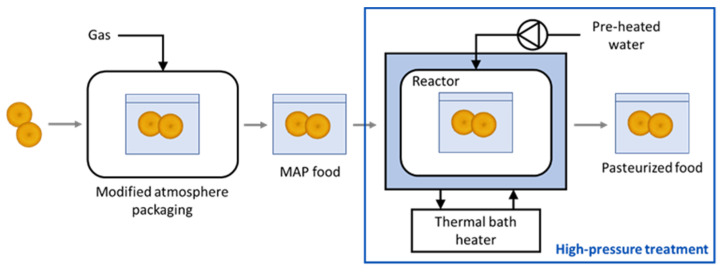
Schematic representation of the ScCO_2_-MAP treatment.

The ScCO_2_-MAP treatment [9] was carried out in two steps (Figure 1): (i) food samples were packaged in CO_2_ MAP and subsequently (ii) subjected to high pressure using water as driving force in a multi-batch apparatus. 

#### 2.3.1. MAP Packaging

MAP packages with an internal chamber of 10 cm × 10 cm and a volume of 100 ± 10 mL were produced from a high gas barrier multilayer (PA/EVOH/PA/PE) film (Euralpack, Schoten, Belgium) with an electrical sealer (PFS-400, Plastic Film Sealer). Preliminary tests (not reported for the sake of conciseness) showed that the packaging material is resistant to high-pressure and supercritical CO_2_, and that these conditions determine neither mechanical damages on the material, nor variations in its barrier properties. Two carrot slices were packaged in each bag. The modified atmosphere was introduced into the packages through a gas mixer (MAP Mix 9001 ME, PBI Dansensor, Segate, Milano, Italy). The sealed packages with the product had a mass of 7.2 ± 0.3 g. To exclude gas leakage or water infiltration during the experiments, the mass and volume of the packages were measured with a scale (PS 6000.R2, Radwag, Radom, Poland) before and after treatment (data not shown). Packages with mass or volume change >5% (unlikely event) were considered damaged and discarded. The gas composition inside the packages was measured with a gas analyzer (Oxybaby M+i O_2_/CO_2_, WITT-Gastechnik GmbH, Witten, Germany). Gas composition was measured before the processing for control samples to ensure a correct gas composition inside the packages, and along the storage to assess the holding of the gas composition inside the packages. Gas composition details are provided in the Supplementary Material.

#### 2.3.2. High Pressure Equipment

The high-pressure treatment was carried out in a multi-batch apparatus (Figure S1). Two steel vessels (R-1 and R-2) with an internal volume of 320 mL each produced replicates under identical process conditions. Vessels are manually loaded at the same time. Each vessel was connected to the equipment piping through an on-off valve (V-1 and V-2, respectively). The vessels R-1 and R-2 were placed in a thermostatic bath to control their temperature. Preheated water was pumped into the vessel by a high-pressure pump (P-1; LDC1, Lewa, Tokyo, Japan). The pressurization was achieved at 4 MPa/min with a water flow rate of 100 mL/min. The system was depressurized at the end of the process by acting on a micrometric valve (V-3; model 2S-4L-N-SS, Rotarex, Brescia, Italy). 

The process temperature was controlled by the thermostatic bath heater (TBH; M408-BC, MPM Instruments s.r.l., Milano, Italy) through the manipulation of a thermal fluid flow inside a copper coil immersed in the thermostatic bath. Pressure was controlled by a pressure controller (PC; ATR241, Pixsys s.r.l., Venice, Italy), which acted on the pump motor. The apparatus was designed to operate at temperatures between 25 and 50 °C, and pressures between 4 and 20 MPa. 

### 2.4. Artificial Vision System

#### 2.4.1. Image Acquisition

**Figure 2:**
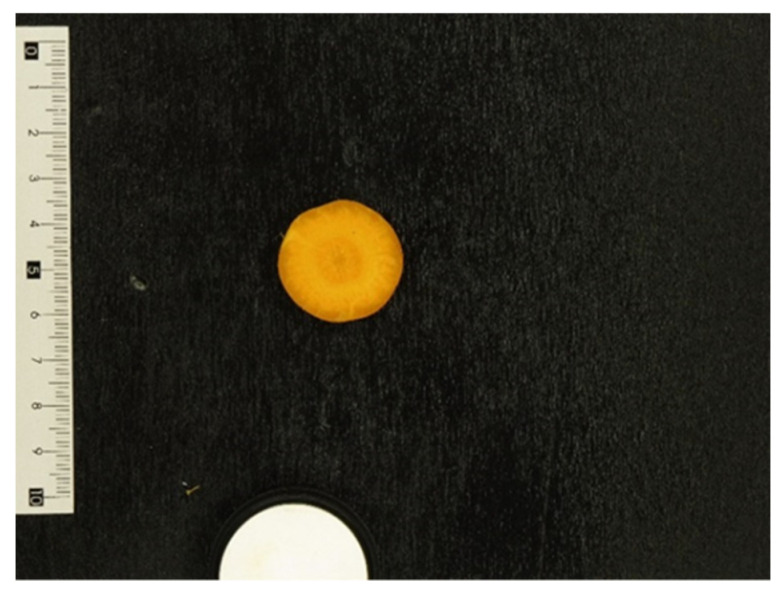
Typical example of image collected by the acquisition apparatus: the carrot sample, the white reference, the black reference, and the dimensional indicator are present in the image scene.

Unpackaged carrot images were collected with an internally developed acquisition apparatus [22] consisting of a box, which shades environmental light, equipped with an illumination system of 4 LED lamps (12V, 4000K, V-Tac, Sofia, Bulgaria) on the four sides of the box to provide uniform illumination. Images were taken with a commercial digital camera (Lumix TZ57 16 MP, Panasonic, Osaka, Japan) fixed on a horizontal support 50 cm above the bottom of the acquisition apparatus. The camera was set with a diaphragm aperture of f/6.3, an exposure time of 1/80 s, and an ISO sensibility of 200. The scene in the collected images contains a food sample (i.e., carrot slice), a white PFA standard reflectance reference (Ocean Optics Inc., Dunedin, FL, USA), a black background, and a dimensional indicator. A typical image used in this analysis is reported in Figure 2.

The collected RGB (red, green, blue) images, $\underline{I}\;\left[J\times K\times C\right]$, had a dimension of 3456 pixels × 4608 pixels × 3 color channels, where 1 pixel has the linear dimension of 33 ± 1 µm. 

Images were taken separately for each carrot slice in the package: (i) at fresh condition 5 min prior the packaging and (ii) at treated condition immediately after unpackaging. Accordingly, O = 168 × 2 = 336 fresh, O = 336 treated, and $O_{c}\;$= 36 control images were analyzed in this study. Hence, 708 carrot images were analyzed. 

#### 2.4.2. Image Analysis

Images were analyzed with an in-house developed software implemented in MATLAB^®^ 2019b (Matworks, Natick, MA, USA) through Image Processing Toolbox (Matworks, Natick, MA, USA), and PLS Toolbox 8.7 (Eigenvector Research Inc., Wenatchee, WA, USA). A three-step procedure was followed: (i) image standardization, (ii) extraction of the region of interest (i.e., carrot sample), and (iii) extraction of features on color and its distribution over the product surface.

Images were initially adjusted [35,36] to compensate for all possible variations caused by lighting conditions and to reconstruct the real color of the image by means of the white and black references that were present in the image scene. 

The region of the original image containing the carrot sample, named the region of interest, was segmented through k-means clustering [37]. The color information was extracted from the region of interest. Then, the largest rectangular portion of the region of interest was identified and used to extract features on the color spatial distribution (which defines the structural characteristics of products). 

The features of the color and its spatial distribution were extracted from each image. $M_{C}$ = 12 color features were extracted as 4 statistical indices of the light intensity distribution of each color channel of an RGB image [22]: mean, standard deviation, skewness, and kurtosis. The features describing the spatial distribution of color were extracted through two different methods: (i) a wavelet texture analysis and (ii) a gray-level co-occurrence matrix (GLCM) from the gray-scale version $I_{g}\;\left[J\times K\right]$ of the original image $\underline{I}$ [J×K×C]. Wavelet texture analysis [29,36,38,39] is a multi-resolution analysis which extracts $M_{W}\;$= 144 structural features (as statistical indices) at different resolutions through the convolution of the original image with orthonormal bases, generated by translation and dilation of the mother wavelet. GLCM [36,39,40,41] uses a probabilistic approach to extract $M_{G}$ = 2420 structural features by considering the relation in space of the pixels of a grayscale image. Details on the implementation of the image analysis software are reported in the Appendix A. 

Features of the color and its spatial distribution extracted from an image were placed in a horizontal vector x [1×M] (M = $M_{C}$ + $M_{W}$ + $M_{G}$), then features from all images were stacked in a vertical manner in order to create a predictor matrix. Features extracted from fresh carrots were organized in the matrix $X_{f}\;\left[O\times M\right]$ = [336 × 2576], while features extracted from all treated and stored images were organized in the matrix $X_{t}\;\left[O\times M\right]$.

#### 2.4.3. Multivariate Hypothesis Testing for Appearance Characterization

Principal component analysis (PCA) [42] was used to test in a multivariate fashion if the treated or stored product preserves a fresh-like visual appearance through multivariate statistical control charts. PCA is a multivariate statistical method that compresses the information in the multivariate dataset of the M = 2576 color-structural features by projecting it into a reduced space of the A orthogonal (i.e., independent) principal components (PCs), which define the direction of maximum variability of the data. 

Once calibrated on fresh samples, PCA provides two statistical indices to judge the appearance of new analyzed product samples: (i) the squared prediction error Q and (ii) Hotelling’s $T^{2}$. Q represents the mismatch between the new analyzed samples and the fresh reference, while $T^{2}$ describes the deviation of each sample from the average color-structural features on which the PCA model is built. Confidence limits for both the residual ($Q_{\lim}$) and Hotelling’s $T^{2}$ ($T_{\lim}^{2}$) can be used to verify whether a new observation could be adequately described by the model and is close to the average conditions (i.e., its appearance is similar to the one of the fresh product) or if it does not conform to the standard conditions in terms of the multivariate correlation structure among features of color and/or its spatial distribution. The 95% confidence limits for both Q and *T*^2^ are defined as

(1) $$Q_{\lim}=\left[\frac{\nu }{2\mu }\right]\chi_{\frac{2\mu^{2}}{v},\alpha }^{2},$$

and

(2) $$T_{\lim}^{2}=\frac{A\left(O-1\right)}{\left(O-A\right)}F_{A,\;O-A,\alpha },$$

where μ and ν are the mean and standard deviation of the Q residuals for the calibration dataset, α is the confidence limit (usually set at 95%), $\chi^{2}$ is a chi-squared distribution, and F represents a F-distribution. Details on PCA are reported in the Appendix B.

In this study, a PCA model with A = 7 PCs was calibrated on features of color and its spatial distribution from fresh carrots $X_{f}$. Accordingly, the multivariate control chart $T^{2}$ vs. Q was used to conduct a hypothesis testing on the carrot appearance. Specifically, $T_{o}^{2}$ and $Q_{o}$ (the statistics for a treated sample o from $X_{t}$) were used to test the null hypothesis that the visual appearance of the treated carrot conforms to the one of fresh products with 95% confidence. If both $T_{o}^{2}\;$<$\;T_{\lim}^{2}$ and $Q_{o}\;$<${\;Q}_{\lim}$, we do not reject the null hypothesis that the treated sample conforms to the fresh vegetables with a confidence of 95%, meaning that the treated carrot cannot be distinguished from fresh products by the artificial vision system. Hence, it is likely that the human eye cannot perceive any differences between fresh and treated product. Conversely, if one of the two statistics $T_{o}^{2}$ and $Q_{o}$ exceeds the respective confidence limits, the null hypothesis is rejected, meaning that the appearance of the treated sample does not conform to that of the fresh food with 95% confidence. In this case, the treated sample is subject to a machine detectable change in color and/or its spatial distribution and has a visual appearance that differs from 95% of the analyzed fresh carrots. Furthermore, the control chart indicates the extent of color and structural changes based on how large the deviations out of the confidence limits are. In fact, since $T_{o}^{2}$ and $Q_{o}$ quantify how well a sample conforms to the model calibrated on fresh samples, large values of these indices relate to large appearance changes.

### 2.5. Microbiological Analyses

The *Escherichia coli* (Migula) Castellani and Chalmers (ATCC 25922) strain was used to inoculate samples to test the ScCO_2_-MAP performance to inactivate fecal contaminants, following the method described previously [43]. 

Some additional samples were inoculated with 40 µL of *E. coli* suspension (20 µL per each carrot side), to reach an inoculation level of 10^8^ CFU/g [44]. After growing overnight, bacteria were first centrifuged (10 min–4696× *g*) and then pellets were resuspended in phosphate buffer saline (PBS, Oxoid, UK) before inoculation. The *E. coli* suspension was uniformly spread in several droplets (3–5 μL) over the carrot surface through a pipet. After inoculation, the samples were dried under a laminar flow hood for 30 min before packaging. Samples which were not inoculated were also analyzed to enumerate the natural microorganisms (i.e., total mesophilic bacteria, yeasts and molds, and *E. coli*) which were present.

For the enumeration, samples were diluted at a ratio of 1:10 in phosphate buffer saline (PBS, Oxoid, UK) and stomached for 1 min in sterile conditions. Appropriate serial dilutions were inoculated into plate count agar (PCA, Sacco, IT), rose bengal agar (RB, Sacco, IT), and chromogenic C-EC II agar (Sacco, IT), and incubated under one of the following conditions: 30 °C for 3 days, 22 °C for 5 days, or 37 °C for 18 h, to investigate the growth of total mesophiles, yeasts and molds, and *E. coli*, respectively. 

The level of inactivation was determined by evaluating the $\log(N/N_{0})$, where $N_{0}$ (CFU/g) is the number of colony forming units present in the untreated sample and N (CFU/g) is the number of survivors after the treatment.

### 2.6. Statistical Tests of the Microbiological Analyses

Analysis of variance (ANOVA) [30] was used for the statistical interpretation of the microbial analysis to assess the effect of pressure, temperature, and treatment time on the microbial population. Effects were considered significant if the ANOVA *p*-value was p ≤ 0.05, while effects were not considered statistically significant otherwise.

## 3. Results and Discussion

This Section shows the results on (i) the selection of the best MAP for untreated fresh carrots; (ii) the identification of the ScCO_2_-MAP operating conditions preserving the fresh appearance of carrots after treatment and evaluation of the appearance change during storage; and (iii) the verification of the microbiological safety for carrots processed at optimal ScCO_2_-MAP operating conditions only.

### 3.1. Identification of the Atmosphere Composition Preserving Fresh-like Product Appearance

**Figure 3:**
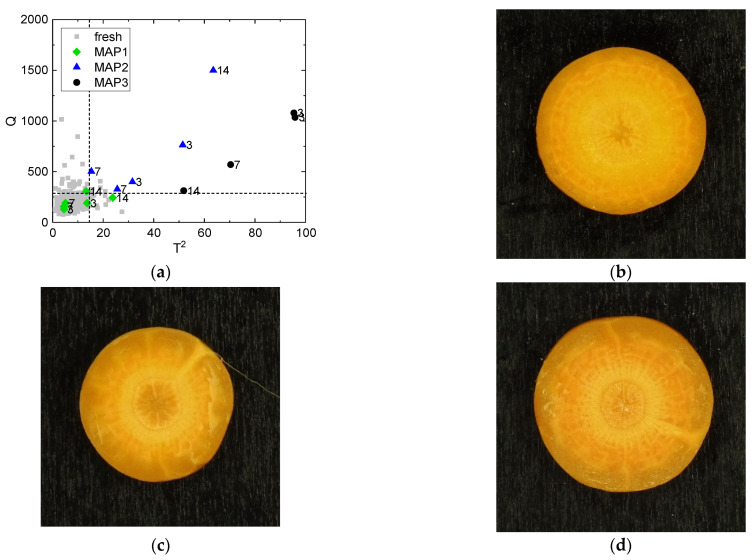
(**a**) Control chart: characterization of the visual changes in terms of color and its spatial distribution during storage at 3, 7, and 14 days for untreated samples packaged with different atmosphere compositions. Fresh samples (fresh)—gray squares; 100% CO_2_ MAP (MAP1)—green diamonds; 90% CO_2_ with 10% O_2_ MAP (MAP2)—blue triangles; air MAP (MAP3)—black dots. The numbers represent the storage time in days. The dashed lines are the 95% confidence limits for $T_{\lim}^{2}$ and $Q_{\lim}$. (**b**) Example of untreated control carrot packaged in MAP1 after 14 days of storage. (**c**) Example of untreated control carrot packaged in MAP2 after 3 days of storage. (**d**) Example of untreated control carrot packaged in MAP3 after 3 days of storage.

The effect of the modified atmosphere composition on the product appearance during storage was preliminarily evaluated to identify which was the optimal MAP composition between MAP1, MAP2, and MAP3 for untreated carrots (i.e., stored samples without being processed with ScCO_2_-MAP) and to validate the multivariate method. For this purpose, multivariate hypothesis testing was performed on all the untreated control samples by projecting them into the PCA model built on fresh carrot samples $X_{f}$ (Section 2.4.3). The control chart (Figure 3a) reports Hotelling’s $T^{2}$ on the x-axis and the residual Q on the y-axis, while the dashed lines are the 95% confidence limits $T_{\lim}^{2}$ and $Q_{\lim}$. The pairs ($T_{o}^{2}$, $Q_{o}$) related to all $X_{f}$ observations (for *o* = 1, 2, …, O) are plotted in the control chart as gray squares and serve as a reference of fresh conditions, while untreated control samples are plotted as colored shapes. The null hypothesis was rejected for all samples packaged in MAP2 and MAP3 (90% CO_2_ with 10% O_2_ and air, respectively) independently of the storage time, because the blue triangles and the black circles are located outside the 95% confidence limits. This indicates that the appearance of the untreated product packaged in the presence of O_2_ (MAP2 and MAP3) did not conform to that of the fresh carrots and appearance changes occurred during storage. An example of what can be visually perceived for carrots packaged and stored in MAP2 and MAP3 after 3 days of storage is reported in Figure 3c,d. Conversely, the null hypothesis was not rejected for the untreated samples packaged in MAP1 (100% CO_2_) up to 7 days of storage (green diamonds in Figure 3a). This means that samples packaged in MAP1 are not distinguishable from fresh carrots with 95% confidence after 7 days of storage. Instead, the null hypothesis was rejected for 14-day samples, because both replicates were located outside at least one of the statistical limits. Despite appearance changes being detected by the artificial vision system, these samples were located close to the statistical limits and to the 5% of non-standard fresh samples out of the 95% confidence limits. For this reason, these samples can still be considered hardly distinguishable from fresh carrots by human perception; hence, they can be considered to have a good appearance quality, and would likely be accepted by consumers. An example of what can be visually perceived for carrots packaged in MAP1 after 14 days of storage is reported in Figure 3b.

The analysis of the atmosphere composition for control samples confirmed that carrots were packaged with the correct MAP composition, and that this composition was correctly preserved in the packages up to 14 days (Supplementary Material, Figures S2 and S3). Only small variations in the O_2_ concentration were found due to instrumental measurement error and tolerances in the MAP filling.

Our findings support the fact that the modified atmosphere composition has an effect on the appearance of carrots during storage [45,46], which are usually subject to surface whitening due to dehydration and lignification [47]. Furthermore, as previously observed [48], the results of MAP2 and MAP3 support the fact that low O_2_ (~10%) has a negative effect on product quality, probably due to cellular respiration and enzymatic reactions. However, further tests should be performed to support these hypotheses and to determine the cause of appearance changes in MAP with low O_2_ concentration.

### 3.2. Identification of the Processing Condition Preserving the Fresh-like Product Appearance

The optimization of the ScCO_2_-MAP preservation process is intended to identify the treatment conditions that minimally affect the visual appearance of the products during storage. The analysis was performed for all treated carrots packaged with three MAPs (MAP1, MAP2, and MAP3) from $X_{t}$ by projecting them into the PCA model built on fresh carrot samples $X_{f}$ (Section 2.4.3). The results confirmed that 100% CO_2_ MAP (MAP1) induces a minimal effect on the visual appearance after the process and during storage, similar to what was presented in Section 3.1. For this reason, only the results obtained for MAP1 are presented and discussed. Results on MAP2 and MAP3 can be found in the Supplementary Material (Figures S5–S7). 

**Figure 4:**
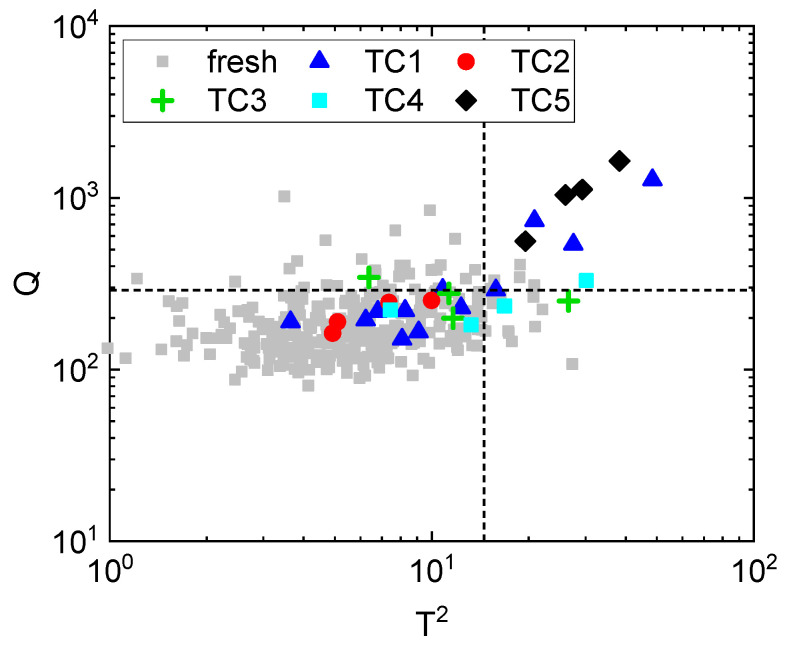
Control chart: characterization of the visual changes just after treatment in terms of color and its spatial distribution for samples treated at different conditions in 100% CO_2_ (MAP1). Fresh samples (fresh)—gray squares; 32.5 °C, 9 MPa, 30 min (TC1)—blue triangles; 40 °C, 6 MPa, 15 min (TC2)—red dots; 25 °C, 6 MPa, 45 min (TC3)—green crosses; 25 °C, 12 MPa, 15 min (TC4)—cyan squares; 40 °C, 12 MPa, 45 min (TC5)—black diamonds. The dashed lines are the 95% confidence limits for $T_{\lim}^{2}$ and $Q_{\lim}$.

The effect of the process conditions was analyzed after treatment (0 days of storage) to identify the conditions that preserve a fresh appearance. The control chart built for these samples is reported in Figure 4, where fresh carrots from $X_{f}$ are plotted as gray squares, and the dashed lines are the 95% confidence limits $T_{\lim}^{2}$ and $Q_{\lim}$. The null hypothesis was rejected for all samples treated at TC5 (40 °C, 12 MPa, 45 min; black diamonds), which were located outside the statistical limits $T_{\lim}^{2}$ and $Q_{\lim}$, and were the most distant samples from the 95% confidence limits. Consequently, TC5 was the most severe treatment and caused a change in appearance with respect to fresh carrots. Similarly, TC3 (25 °C, 6 MPa, 45 min; green crosses) and TC4 (25 °C, 12 MPa, 15 min; cyan squares) produced appearance changes that oftentimes made the samples distinguishable from fresh carrots; in fact, for both TC3 and TC4 the null hypothesis was rejected for 50% of the samples (2 over 4 samples), which were located outside the statistical limits. On the contrary, 66.6% of the samples treated at TC1 (32.5 °C, 9 MPa, 30 min; blue triangles) remained similar to the fresh carrots after treatment. In fact, the null hypothesis was not rejected in 66.6% of the samples (8 over 12 samples), because they were located inside the statistical limits $T_{\lim}^{2}$ and $Q_{\lim}$. However, a large variability in the appearance of these samples was observed. In fact, three samples treated at TC1 showed a very high appearance change, compatible with a more severe treatment condition (that is, TC5). Samples treated at TC2 (40 °C, 6 MPa, 15 min; red dots) could not be distinguished from fresh carrots by the artificial vision system (with 95% confidence), because the null hypothesis was not rejected for all the samples (4 over 4 samples). Accordingly, TC1 and TC2 are the most promising conditions for preserving the fresh appearance of RTE carrots after ScCO_2_-MAP treatment.

These results show that very high pressure (12 MPa) and long treatment time (45 min) have a stronger impact on carrot appearance than high temperature (40 °C). The influence of longer treatment times on carrots’ color has previously been observed by Ferrentino et al. [15]. 

**Figure 5:**
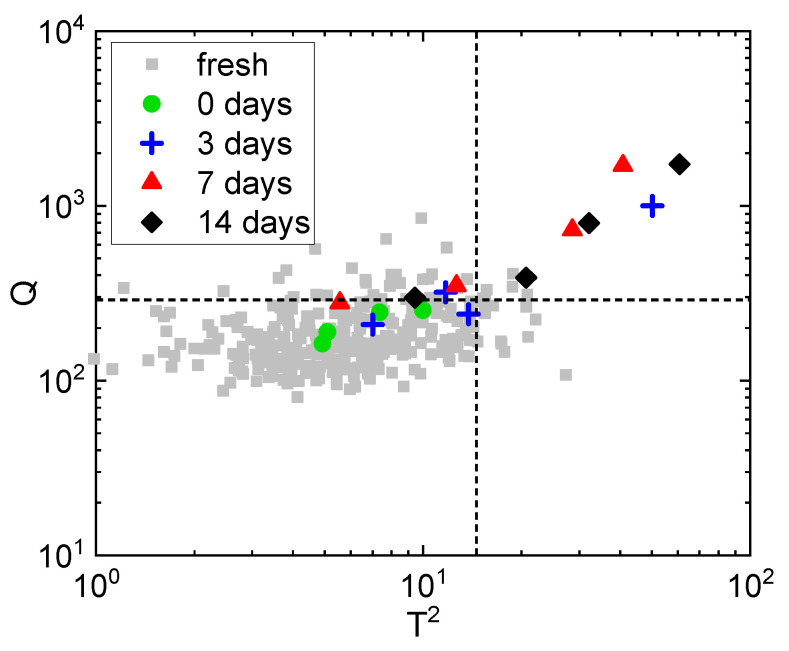
Control chart: characterization of the visual changes in terms of color and its spatial distribution during storage for samples treated at 40 °C, 6 MPa, 15 min (TC2) in 100% CO_2_ (MAP1). Fresh samples (fresh)—gray squares; after treatment (0 days of storage)—green dots; 3 days of storage—blue crosses; 7 days of storage—red triangles; 14 days of storage—black diamonds. The dashed lines are the 95% confidence limits for $T_{\lim}^{2}$ and $Q_{\lim}$.

**Figure 6:**
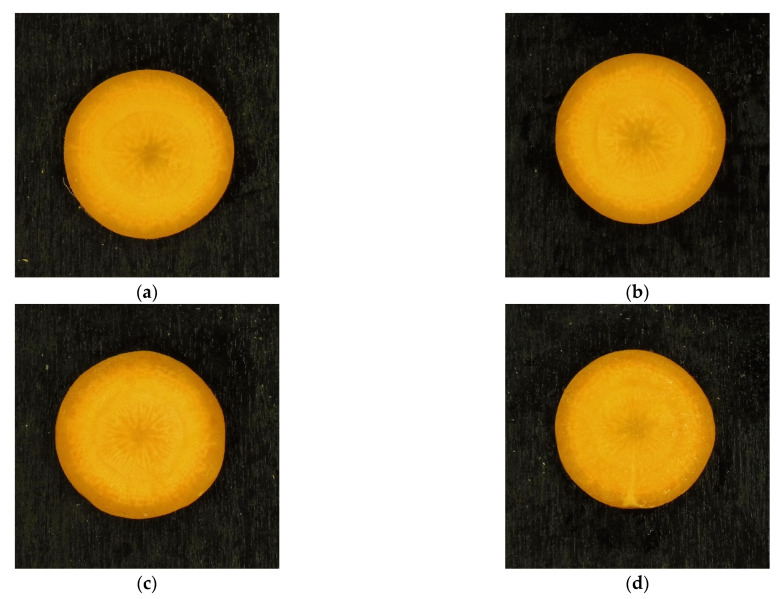
Example of carrot images for comparison: (**a**) fresh, (**b**) TC2 (40 °C, 6 MPa, 15 min) after treatment, (**c**) TC2 after 7 days of storage, (**d**) TC2 after 14 days of storage. Carrots were packaged in 100% CO_2_ (MAP1).

According to the above-mentioned result, the control chart for samples treated at TC2 during 14 days of storage was considered to understand the stability of the appearance over time (Figure 5). At 3 days of storage (blue crosses), the null hypothesis was not rejected in 50% of samples (2 over 4 samples,); however, for one of the samples the null hypothesis was rejected even if it was located very close to the statistical limits, meaning that this sample was subject only to marginal appearance changes. At 7 days of storage (red triangles), the null hypothesis was rejected for 75% of samples (3 over 4 samples), indicating that the majority of the samples showed some variation in color and its spatial distribution. Moreover, the null hypothesis was rejected for all samples (4 over 4 samples) at 14 days of storage (black diamonds), which were located outside the statistical limits. In summary, carrots treated at 40 °C, 6 MPa, and 15 min, and stored at 4 °C are often not distinguishable from fresh carrots in the initial days of storage, while after 7–14 days the appearance change from the fresh product of these samples is evident. An example of the appearance change that can be visually perceived during storage for carrots treated at TC2 is reported in Figure 6a–d. However, the extent of these appearance changes (i.e., the distance from the statistical limits $T_{\lim}^{2}$ and $Q_{\lim}$) was often small compared to the appearance changes produced by heavier treatment conditions (i.e., higher pressures) as shown in Figure 3. For example, the treatment at 40 °C, 12 MPa, and 45 min (TC5) alone generally produced larger appearance changes compared to carrots stored for 14 days after being processed at 40 °C, 6 MPa, and 15 min (TC2). This result indicates that high pressure (12 MPa) produces a larger effect on appearance than the combined effect of lower pressure (6 MPa) and 14 days of storage. 

Samples treated at TC1 (32.5 °C, 9 MPa, 30 min) with a storage time ranging from 3 to 14 days were distinguishable from fresh carrots (Supplementary Material, Figure S4), because the null hypothesis was rejected for 91.7% of samples (11 over 12 samples). In this case, even if the treatment itself produced moderate appearance changes, the higher pressure and longer treatment time produced modifications in the vegetable matrix that caused a larger appearance change during storage. However, additional studies should be performed to understand which factor among temperature, pressure, and treatment time mostly affect the appearance stability during storage. 

The analysis of the atmosphere composition (Supplementary Material, Figures S2 and S3) confirmed that the atmosphere composition defined in the experimental plan was maintained in the above-mentioned conditions up to 14 days of storage.

### 3.3. Microbial Analyses

Microbial analysis was performed only under the processing conditions identified by the artificial vision system (in Section 3.2) that ensure the preservation of the fresh appearance of RTE carrots. Accordingly, samples packaged in MAP1 and treated at TC1 (32.5 °C, 9 MPa, 30 min) and TC2 (40 °C, 6 MPa, 15 min) were tested for microbial safety, although TC1 did not provide a good preservation of product appearance during storage, while TC2 did. Nevertheless, the former was also analyzed to compare the inactivation performance of CO_2_ at both supercritical (TC1) and subcritical conditions (TC2).

The detected natural flora of untreated fresh-cut carrots was composed of 5.44 ± 0.20 log (CFU/g) mesophilic microorganisms and 4.90 ± 0.22 log (CFU/g) yeasts and molds, in accordance with other analyses performed on fresh-cut carrots [11]. No *E. coli* colonies were detected within the natural microbiota on the surface of the carrots. 

**Figure 7:**
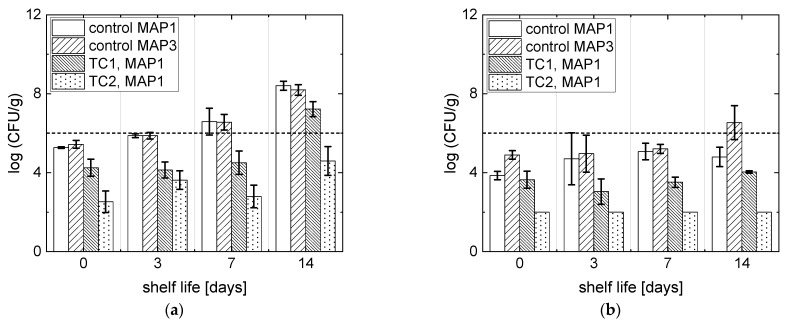
Microbial population profile of fresh cut carrots during storage: (**a**) mesophiles and (**b**) yeasts and molds. The dotted line indicates the 6 log (CFU/g) threshold to define a sample spoiled. Untreated stored sample (control); 100% CO_2_ (MAP1); air (MAP3); 32.5 °C, 9 MPa, 30 min (TC1); 40 °C, 6 MPa, 15 min (TC2).

The microbial population of fresh-cut carrots during shelf life in terms of mesophilic microorganisms is reported in Figure 7a. Control samples packaged in MAP1 and MAP3 exhibited a steady growth in the population of mesophilic microorganisms during shelf life, and after 1 week they both reached >6 log (CFU/g) for mesophilic microorganisms, usually considered a threshold value by the food industry. For untreated control samples, ANOVA indicates that the atmosphere composition did not have an impact on the microbial population during shelf life (p > 0.05), meaning that CO_2_ itself is not able to inhibit the growth of total mesophilic bacteria, nor does the presence of oxygen promote microbial proliferation. All the analyzed treated samples exhibited an initial inactivation >1 log (CFU/g): specifically, TC1 induced 1.2 log (CFU/g), while a higher inactivation of 2.9 log (CFU/g) was achieved with TC2. Only the carrots treated at TC2 were able to maintain the total microbial load <6 log (CFU/g) up to 14 days, while at less than 14 days of storage they were able to induce a substantial growth after processing at TC1. In these cases, additional tests would be required to accurately determine the actual shelf life of the products. ANOVA indicates that TC1 and TC2 provided a significantly different inactivation of the microbial population (p < 0.001) and showed a significantly different microbial growth profile during shelf life (p < 0.001). Accordingly, TC1 provides a larger inactivation performance and a longer stability over storage time. 

The microbial population of carrots during shelf life in terms of yeasts and molds is reported in Figure 7b. Control samples packaged in air (MAP3) showed a steadily growing profile in yeasts and molds population, and the total population of yeasts and molds was >6 log (CFU/g) at 14 days of shelf life. Control samples packaged in MAP1, instead, showed a significant reduction in the population of yeasts and molds identified with ANOVA (p < 0.001), and the population remained stable up to 14 days. This indicates that yeasts and molds are more sensitive to the CO_2_ than mesophilic bacteria as previously observed [49], and the absence of oxygen reduces the growth of yeast and mold populations. The products treated at TC2 showed the highest inactivation for yeasts and molds, which resulted in them being below the detectability limits of the technique (<100 CFU/g) during the whole shelf life. However, a certain degree of inactivation was also achieved with treatment TC1, which inhibited microorganisms’ growth during storage. TC1 and TC2 showed statistically different inactivation performances for the entire shelf life according to ANOVA (p < 0.001). 

According to these results, the combination of high temperature (40 °C) and pressurized CO_2_ determines a more efficient inactivation than supercritical CO_2_ at low temperature (32.5 °C).

The inactivation with ScCO_2_-MAP was also tested on inoculated *E. coli*. A 1.98 ± 0.48 log (CFU/g) and 2.27 ± 0.32 log (CFU/g) inactivation were achieved with treatments TC1 and TC2, respectively. Furthermore, no significant differences in inactivation performance (ANOVA p > 0.05) were found between TC1 and TC2, indicating that the achieved *E. coli* inactivation is independent of the specific treatment condition, indicating that neither higher temperature (40 °C) nor supercritical CO_2_ provide higher performance in *E. coli* inactivation. This finding indicates that the ScCO_2_-MAP preservation process provides different inactivation performances for different microbial species (i.e., mesophilic microorganisms, yeasts, molds, and *E. coli*) as previously seen with the conventional ScCO_2_ treatment [50]. However, the process induces a 99% reduction in the initial *E. coli* population, thus increasing the product safety against fecal contamination.

## 4. Conclusions

In this study, we optimized the processing conditions of an innovative high-pressure CO_2_ preservation process to reduce the appearance change during the maintenance of cut carrots at 4 °C using an artificial vision system. The developed methodology was able to characterize the appearance changes in terms of color and superficial structure and provided an easy and fast identification of the products with a fresh-like appearance, which will be easily accepted by consumers. 

A 100% CO_2_ modified atmosphere packaging alone was able to the preserve the visual appearance of ready-to-eat carrots, but it does not guarantee microbial stability. Processing in 100% CO_2_ MAP at 40 °C, 6 MPa, and 15 min maintained the fresh appearance of carrots up to 7 days of storage, providing a 2.9 log (CFU/g) inactivation and a microbiologic stability over 14 days of storage at 4 °C. The process was able to inactivate 2 log (CFU/g) of inoculated *E. coli* on the surface. However, in the carrots after 7–14 days, perceivable appearance changes were detected; additional studies should be performed to demonstrate the acceptability of the product through panel/consumer testing. Moreover, tests on other sensorial and chemical characteristics, such as flavor, texture, humidity, and nutrient content, should be performed in future studies. 

The developed artificial vision system has the potential to be applied to any food treatment involving changes in the appearance of products. Furthermore, future investigations should consider the development of an artificial vision system capable of directly analyzing the packaged product, which can be useful as routine qualitative tests.

## Figures and Tables

**Table 1:** Plan of treatment conditions selected by the 2^3−1^ fractional factorial DoE. Note that TC1 corresponds to the central point of the 2^3−1^ fractional factorial DoE.

Name	Temperature (°C)	Pressure (MPa)	Treatment Time (min)
TC1	32.5	9	30
TC2	40	6	15
TC3	25	6	45
TC4	25	12	15
TC5	40	12	45

## Data Availability

The data presented in this study are available on request from the corresponding author. The data are not publicly available due to privacy reasons.

## Supplementary Materials

The following are available online at https://www.mdpi.com/article/10.3390/foods10122999/s1, Figure S1: ScCO_2_-MAP equipment; Figure S2: MAP CO_2_ composition; Figure S3: MAP O_2_ composition; Figure S4: MAP1 TC1 shelf life; Figure S5: MAP2 treatment conditions; Figure S6: MAP2 TC2 shelf life; Figure S7: MAP3 treatment conditions.

## Appendix A. Artificial Vision System Details

Wavelet texture analysis [29,38,39] extracts information at different resolutions through the convolution of the original image with orthonormal bases, generated by translation and dilation of a Coilfet mother wavelet with three vanishing moments. In particular, at each resolution H = 4 sub-images (one approximation and three details) are generated: horizontal, vertical, and diagonal high-frequency information in the details, and low-frequency information in the approximation. A total of R = 6 resolution scales are analyzed convolving the approximation sub-image of the previous resolution scale, while at resolution r = 1 the original image is used. At each resolution scale six statistical descriptors are extracted from the distribution of the pixel light intensities of all the four sub-images: energy, mean, standard deviation, skewness, kurtosis, and entropy [22,36,51].

GLCM [40] considers the relation in space of the pixels of a grayscale image to extract information on the spatial distribution of color over the image surface. In GLCM, the spatial relation between two pixels, located at different distances and directions, is analyzed by moving from one pixel in both the senses of the pixel rows j and the pixel columns k of the distance vectors d = [1 2 3 5 10 20 30 40 50 60 65] and -d, thus generating a D = 484 combination. These combinations are selected to obtain a resolution scale of GLCM comparable with the one of the wavelet decomposition. Furthermore, in GLCM the entire range of light intensity is discretized in 64 bins to reduce the number of possible combinations among light intensities [41]. Features of the spatial distribution of color are five statistical descriptors: contrast, correlation, energy, homogeneity, and entropy [39,40,51].

## Appendix B. Principal Components Analysis

Principal component analysis (PCA) [42] is a multivariate statistical method which compresses the information in the autoscaled (i.e., centered to the mean and scaled to unit variance) dataset $X_{f}\;\left[O\times M\right]$, by projecting it into a reduced space of A orthogonal (i.e., independent) principal components (PCs), defining the direction of maximum variability of the dataset. In this paper, the proper number of PCs is selected through a scree test. The projection onto the low-dimensional space of the *A* PCs is performed according to

(A1) $$X=TP^{T}+E,$$

where T [O×A], P [A×M], and E [O×M] are the score, the loading, and the residual matrices, respectively, and the superscript T indicates the matrix transpose. The score matrix contains information on how the O observations are related, the loading matrix contains information on the correlation structure among the M color-spatial features of the input dataset, while the residual matrix E is minimized and contains the amount of information not included into the A selected PCs of the model.

Any new observation $x_{o}$ (i.e., either a generic index for the *O* matrix rows or a new observation) can be projected into the PCA model according to

(A2) $$t_{o}=x_{o}P,$$

where $t_{o}\;\left[1\times A\right]$ is the score vector of the new observation.

The residuals of the observation *o* can be summarized in the squared prediction error, $Q_{o}$, which represents the mismatch between the original data and their PCA reconstruction:

(A3) $$Q_{o}=e_{o}e_{o}^{T},$$

where $e_{o}\;\left[1\times M\right]$ is the residual vector of the observation $x_{o}$ obtained from

(A4) $$e_{o}=x_{o}-t_{o}P^{T},$$

where $t_{o}$ is the score vector of the observation o calculated by projecting $x_{o}$ into the PCA model. Similarly, the sample deviation from the average observation is described by means of Hotelling’s $T^{2}$ statistics:

(A5) $$T_{o}^{2}=x_{o}P\Lambda^{-1}P^{T}x_{o}^{T},$$

where $\Lambda^{-1}$ is a [A × A] diagonal matrix containing the inverse eigenvalues associated to the A PCs.

## References

[1] Davis W., Lucier G. (2021). Vegetable and Pulses Outlook: April 2021.

[2] Müller L., Kjelsø C., Frank C., Jensen T., Torpdahl M., Soborg B., Dorleans F., Rabsch W., Prager R., Gossner C.M. (2016). Outbreak of Salmonella Strathcona caused by datterino tomatoes, Denmark, 2011. Epidemiol. Infect..

[3] Vestrheim D.F., Lange H., Nygard K., Borgen K., Wester A.L., Kvarme M.L., Vold L. (2016). Are ready-to-eat salads ready to eat? An outbreak of Salmonella Coeln linked to imported, mixed, pre-washed and bagged salad, Norway, November 2013. Epidemiol. Infect..

[4] Tejedor-Calvo E., Morales D., García-Barreda S., Sánchez S., Venturini M.E., Blanco D., Soler-Rivas C., Marco P. (2020). Effects of gamma irradiation on the shelf-life and bioactive compounds of Tuber aestivum truffles packaged in passive modified atmosphere. Int. J. Food Microbiol..

[5] Li L., Li C., Sun J., Xin M., Yi P., He X., Sheng J., Zhou Z., Ling D., Zheng F. (2021). Synergistic effects of ultraviolet light irradiation and high-oxygen modified atmosphere packaging on physiological quality, microbial growth and lignification metabolism of fresh-cut carrots. Postharvest Biol. Technol..

[6] Pinto L., Palma A., Cefola M., Pace B., D’Aquino S., Carboni C., Baruzzi F. (2020). Effect of modified atmosphere packaging (MAP) and gaseous ozone pre-packaging treatment on the physico-chemical, microbiological and sensory quality of small berry fruit. Food Packag. Shelf Life.

[7] Huang Y.C., Yang Y.H., Sridhar K., Tsai P.J. (2021). Synergies of modified atmosphere packaging and high-voltage electrostatic field to extend the shelf-life of fresh-cut cabbage and baby corn. Lwt.

[8] Zhou B., Zhang L., Wang X., Dong P., Hu X., Zhang Y. (2019). Inactivation of Escherichia coli O157:H7 by high hydrostatic pressure combined with gas packaging. Microorganisms.

[9] Spilimbergo S., Zambon A., Michelino F., Polato S. (2020). Method for Food Pasteurization. U.S. Patent.

[10] Bi X., Wu J., Zhang Y., Xu Z., Liao X. (2011). High pressure carbon dioxide treatment for fresh-cut carrot slices. Innov. Food Sci. Emerg. Technol..

[11] Spilimbergo S., Komes D., Vojvodic A., Levaj B., Ferrentino G. (2013). High pressure carbon dioxide pasteurization of fresh-cut carrot. J. Supercrit. Fluids.

[12] Oey I., Lille M., Van Loey A., Hendrickx M. (2008). Effect of high-pressure processing on colour, texture and flavour of fruit- and vegetable-based food products: A review. Trends Food Sci. Technol..

[13] Zudaire L., Lafarga T., Viñas I., Abadias M., Brunton N., Aguiló-Aguayo I. (2019). Effect of Ultrasound Pre-Treatment on the Physical, Microbiological, and Antioxidant Properties of Calçots. Food Bioprocess Technol..

[14] Zhou L., Bi X., Xu Z., Yang Y., Liao X. (2015). Effects of High-Pressure CO_2_ Processing on Flavor, Texture, and Color of Foods. Crit. Rev. Food Sci. Nutr..

[15] Ferrentino G., Balzan S., Spilimbergo S. (2012). On-line color monitoring of solid foods during supercritical CO_2_ pasteurization. J. Food Eng..

[16] Rico D., Martín-Diana A.B., Barat J.M., Barry-Ryan C. (2007). Extending and measuring the quality of fresh-cut fruit and vegetables: A review. Trends Food Sci. Technol..

[17] Yagiz Y., Balaban M.O., Kristinsson H.G., Welt B.A., Marshall M.R. (2009). Comparison of Minolta colorimeter and machine vision system in measuring colour of irradiated Atlantic salmon. J. Sci. Food Agric..

[18] Goñi S.M., Salvadori V.O. (2017). Color measurement: Comparison of colorimeter vs. computer vision system. J. Food Meas. Charact..

[19] León K., Mery D., Pedreschi F., León J. (2006). Color measurement in L*a*b* units from RGB digital images. Food Res. Int..

[20] Pathare P.B., Opara U.L., Al-Said F.A.J. (2013). Colour Measurement and Analysis in Fresh and Processed Foods: A Review. Food Bioprocess Technol..

[21] Boschetti L., Ottavian M., Facco P., Barolo M., Serva L., Balzan S., Novelli E. (2013). A correlative study on data from pork carcass and processed meat (Bauernspeck) for automatic estimation of chemical parameters by means of near-infrared spectroscopy. Meat Sci..

[22] Ottavian M., Fasolato L., Serva L., Facco P., Barolo M. (2014). Data Fusion for Food Authentication: Fresh/Frozen-Thawed Discrimination in West African Goatfish (Pseudupeneus prayensis) Fillets. Food Bioprocess Technol..

[23] Cubero S., Aleixos N., Moltó E., Gómez-Sanchis J., Blasco J. (2011). Advances in Machine Vision Applications for Automatic Inspection and Quality Evaluation of Fruits and Vegetables. Food Bioprocess Technol..

[24] Momin M.A., Yamamoto K., Miyamoto M., Kondo N., Grift T. (2017). Machine vision based soybean quality evaluation. Comput. Electron. Agric..

[25] Nandi C.S., Tudu B., Koley C. (2016). A machine vision technique for grading of harvested mangoes based on maturity and quality. IEEE Sens. J..

[26] Kiani S., Minaei S. (2016). Potential application of machine vision technology to saffron (Crocus sativus L.) quality characterization. Food Chem..

[27] Cubero S., Lee W.S., Aleixos N., Albert F., Blasco J. (2016). Automated Systems Based on Machine Vision for Inspecting Citrus Fruits from the Field to Postharvest—A Review. Food Bioprocess Technol..

[28] Jackman P., Sun D.W. (2013). Recent advances in image processing using image texture features for food quality assessment. Trends Food Sci. Technol..

[29] Liu J.J., MacGregor J.F., Duchesne C., Bartolacci G. (2005). Flotation froth monitoring using multiresolutional multivariate image analysis. Miner. Eng..

[30] Montgomery D.C. (2007). Design and Analysis of Experiments: Second Edition. Des. Anal. Exp. Second Ed..

[31] Yu T., Niu L., Iwahashi H. (2020). High-Pressure Carbon Dioxide Used for Pasteurization in Food Industry. Food Eng. Rev..

[32] Fleury C., Savoire R., Harscoat-Schiavo C., Hadj-Sassi A., Subra-Paternault P. (2018). Optimization of supercritical CO_2_ process to pasteurize dietary supplement: Influencing factors and CO_2_ transfer approach. J. Supercrit. Fluids.

[33] Cappelletti M., Ferrentino G., Spilimbergo S. (2014). Supercritical carbon dioxide combined with high power ultrasound: An effective method for the pasteurization of coconut water. J. Supercrit. Fluids.

[34] Garcia-Gonzalez L., Geeraerd A.H., Spilimbergo S., Elst K., Van Ginneken L., Debevere J., Van Impe J.F., Devlieghere F. (2007). High pressure carbon dioxide inactivation of microorganisms in foods: The past, the present and the future. Int. J. Food Microbiol..

[35] Russ J.C. (2011). The Image Processing Handbook.

[36] Facco P., Santomaso A.C., Barolo M. (2017). Artificial vision system for particle size characterization from bulk materials. Chem. Eng. Sci..

[37] Hartigan J.A., Wong M.A. (1979). Algorithm AS 136: A K-Means Clustering Algorithm. Appl. Stat..

[38] Addison P.S. (2016). The Illustrated Wavelet Transform Handbook.

[39] Bharati M.H., Liu J.J., MacGregor J.F. (2004). Image texture analysis: Methods and comparisons. Chemom. Intell. Lab. Syst..

[40] Haralick R.M., Dinstein I., Shanmugam K. (1973). Textural Features for Image Classification. IEEE Trans. Syst. Man Cybern..

[41] Tessier J., Duchesne C., Gauthier C., Dufour G. (2008). Estimation of alumina content of anode cover materials using multivariate image analysis techniques. Chem. Eng. Sci..

[42] Wold S., Esbensen K., Geladi P. (1987). Principal component analysis. Chemom. Intell. Lab. Syst..

[43] González-Alonso V., Cappelletti M., Bertolini F.M., Lomolino G., Zambon A., Spilimbergo S. (2020). Research Note: Microbial inactivation of raw chicken meat by supercritical carbon dioxide treatment alone and in combination with fresh culinary herbs. Poult. Sci..

[44] Zhou Z., Zuber S., Cantergiani F., Sampers I., Devlieghere F., Uyttendaele M. (2018). Inactivation of Foodborne Pathogens and Their Surrogates on Fresh and Frozen Strawberries Using Gaseous Ozone. Front. Sustain. Food Syst..

[45] Alasalvar C., Al-Farsi M., Quantick P.C., Shahidi F., Wiktorowicz R. (2005). Effect of chill storage and modified atmosphere packaging (MAP) on antioxidant activity, anthocyanins, carotenoids, phenolics and sensory quality of ready-to-eat shredded orange and purple carrots. Food Chem..

[46] Esturk O., Ayhan Z., Gokkurt T. (2015). Minimal Processing and Modified Atmosphere Packaging of Carrot Discs: Effects of Packaging Film and Product Weight. Int. J. Food Process. Technol..

[47] Chen C., Hu W., Zhang R., Jiang A., Liu C. (2018). Effects of hydrogen sulfide on the surface whitening and physiological responses of fresh-cut carrots. J. Sci. Food Agric..

[48] Ayhan Z., Eştürk O., Taş E. (2008). Effect of modified atmosphere packaging on the quality and shelf life of minimally processed carrots. Turk. J. Agric. For..

[49] Zambon A., Michelino F., Bourdoux S., Devlieghere F., Sut S., Dall’Acqua S., Rajkovic A., Spilimbergo S. (2018). Microbial inactivation efficiency of supercritical CO_2_ drying process. Dry. Technol..

[50] Ferrentino G., Spilimbergo S. (2011). High pressure carbon dioxide pasteurization of solid foods: Current knowledge and future outlooks. Trends Food Sci. Technol..

[51] Facco P., Tomba E., Roso M., Modesti M., Bezzo F., Barolo M. (2010). Automatic characterization of nanofiber assemblies by image texture analysis. Chemom. Intell. Lab. Syst..

