# Supplementary material for: Optimization of the Appearance Quality in CO2 Processed Ready-to-Eat Carrots through Image Analysis
Source: Foods. 2021 Dec 4;10(12):2999. doi: 10.3390/foods10122999 (PMC8700774; doi:10.3390/foods10122999)
Supplement: Supplementary file 1 [file foods-10-02999-s001.zip › foods-1466040-supplementary.pdf]

Supplementary Material

A. ScCO$_2$-MAP equipment

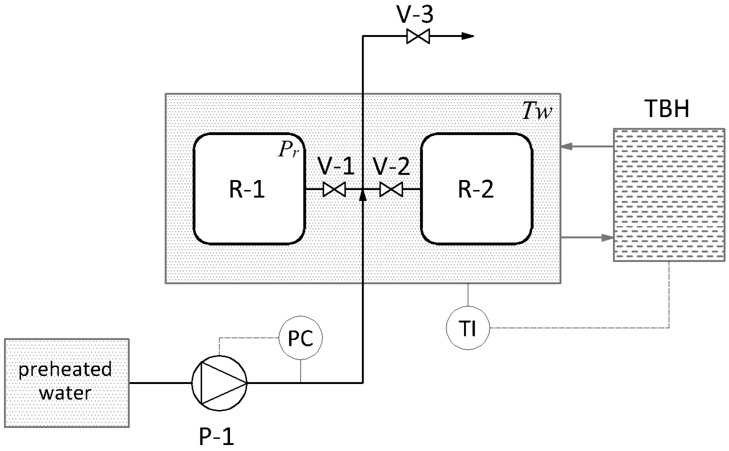

Figure S1 *HPMAP-CO$_2$ plant, containing two steel vessels (R-1 and R-2), two on-off valves (V-1 and V-2), one micrometric valve (V-3), a high-pressure pump (P-1) and a thermostatic bath heater (TBH). The temperature indicator (TI) monitors the temperature of the water in the bath (T_w) and the pressure controller (PC) controls the pressure inside the vessels (P_r).*

B. MAP composition data

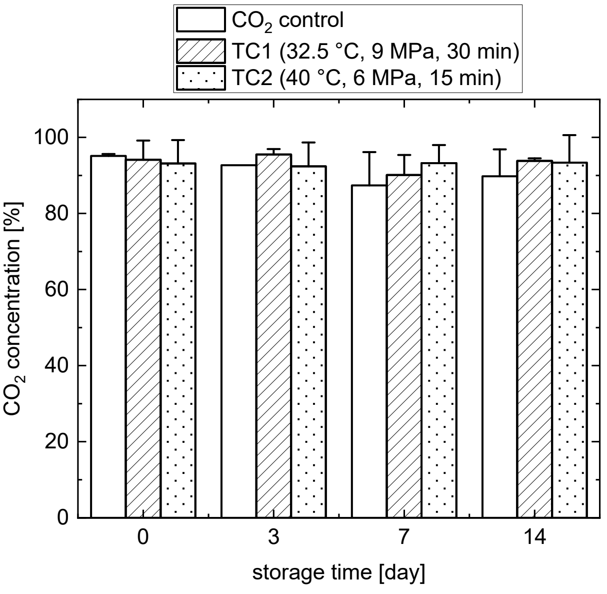

Figure S2 *MAP composition data: CO$_2$ concentration during storage. CO$_2$ control are untreated control samples packaged in 100% CO$_2$, TC1 and TC2 are treated samples packaged in 100% CO$_2$.*

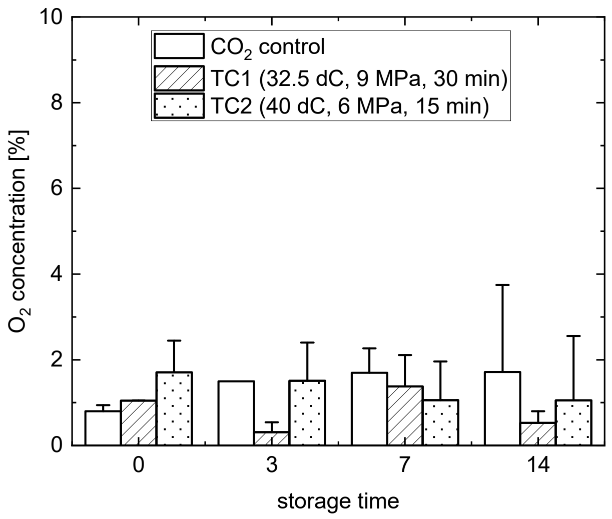

Figure S3 *MAP composition data: O_2 concentration during storage. CO_2 control are untreated control samples packaged in 100% CO_2, TC1 and TC2 are treated samples packaged in 100% CO_2.*

C. MAP1 TC1

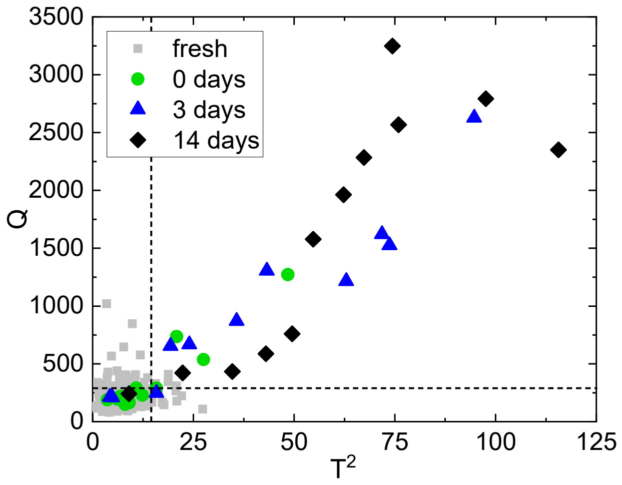

Figure S4 *Control chart: characterization of the visual changes in terms of color and its spatial distribution during storage for samples treated at 32.5 °C, 9 MPa, 30 min (TC1) in 100% CO_2 (MAP1). Fresh samples (fresh) - gray squares; after treatment (0 days of storage) - green dots; 3 days of storage – blue triangles; 14 days of storage – black. The dashed lines are the 95% confidence limits for T^2_{lim} and Q_{lim}. Samples stored for 7 days are omitted for clarity.*

Copyright © 2021 - Barberi, González-Alonso, Spilimbergo, Barolo, Zambon, Facco

D. MAP2

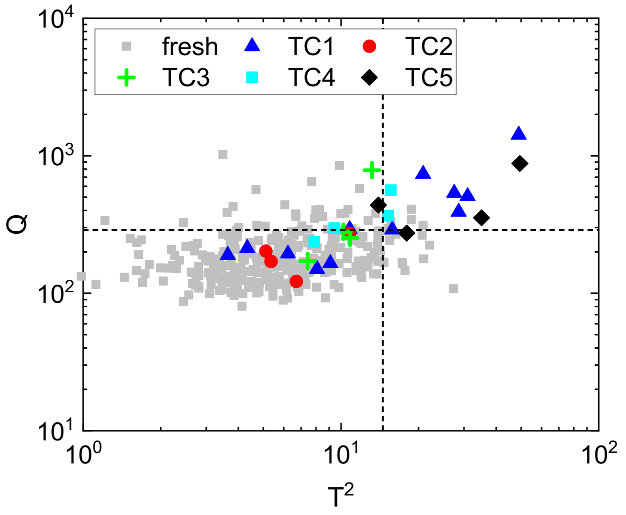

Figure S5 *Control chart: characterization of the visual changes in terms of color and its spatial distribution during storage for samples packaged in 90%* CO_2 *and 10%* O_2 *(MAP2). Fresh samples (fresh) - gray squares; 32.5 °C, 9 MPa, 30 min (TC1) - blue triangles; 40 °C, 6 MPa, 15 min (TC2) – red dots; 25 °C, 6 MPa, 45 min (TC3) – green crosses; 25 °C, 12 MPa, 15 min (TC4) – cyan squares; 40 °C, 12 MPa, 45 min (TC5) – black diamonds. The dashed lines are the 95% confidence limits for* T^2_{lim} *and* Q_{lim}.

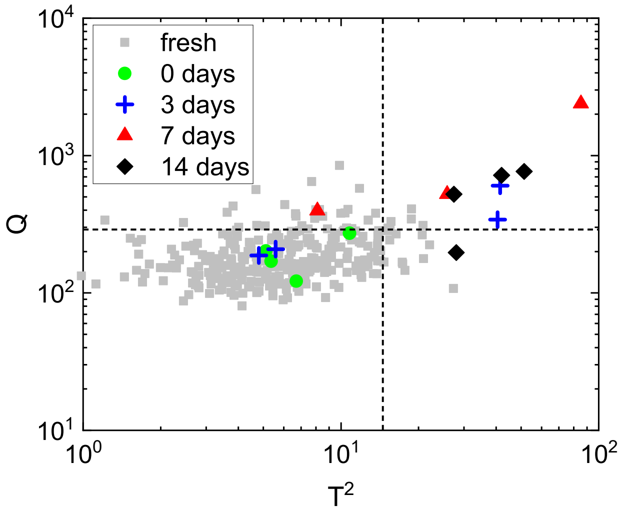

Figure S6 *Control chart: characterization of the visual changes in terms of color and its spatial distribution during storage for samples treated at 40 °C, 6 MPa, 15 min (TC2) in 90%* CO_2 *and 10%* O_2 *(MAP2). Fresh samples (fresh) - gray squares; after treatment (0 days of storage) - green dots; 3 days of storage – blue triangles; 14 days of storage – black. The dashed lines are the 95% confidence limits for* T^2_{lim} *and* Q_{lim}.

Copyright © 2021 - Barberi, González-Alonso, Spilimbergo, Barolo, Zambon, Facco

E. MAP3

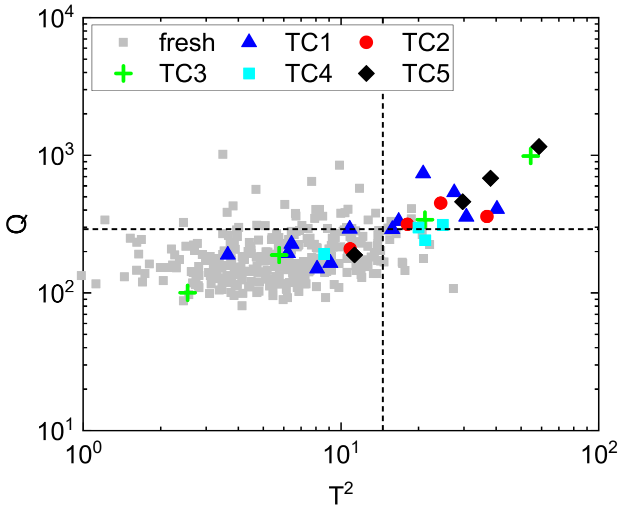

Figure S7 *Control chart: characterization of the visual changes in terms of color and its spatial distribution during storage for samples packaged in air (MAP3). Fresh samples (fresh) - gray squares; 32.5 °C, 9 MPa, 30 min (TC1) - blue triangles; 40 °C, 6 MPa, 15 min (TC2) – red dots; 25 °C, 6 MPa, 45 min (TC3) – green crosses; 25 °C, 12 MPa, 15 min (TC4) – cyan squares; 40 °C, 12 MPa, 45 min (TC5) – black diamonds. The dashed lines are the 95% confidence limits for* T^2_{lim} *and* Q_{lim}.

Copyright © 2021 - Barberi, González-Alonso, Spilimbergo, Barolo, Zambon, Facco
